# Clinical Audit of Psychiatric Discharge Summaries Complying with STOMP (Stopping Over Medication Of People With a Learning Disability and Autism) from Learning Disability Services

**DOI:** 10.1192/bjo.2025.10589

**Published:** 2025-06-20

**Authors:** Rebecca Fernandes, Claire Donnelly, Padma Babu

**Affiliations:** TEWV NHS Foundation Trust, Durham, United Kingdom

## Abstract

**Aims:** Long-term administration of psychotropic medications can be associated with significant side effects and physical health problems. There is evidence that people with intellectual disability have overall poorer health than their non-disabled peers. Psychotropic medication prescribed must be reviewed regularly to avoid routine continuation. NICE Challenging behaviour and learning disabilities [NG11] recommends medication should be initiated by a specialist and they should record:

The rationale for medication and the likely length of treatment.

A written strategy for reviewing and stopping the medication that must be shared with non-specialist colleagues.

The Learning disability teams within Tees, Esk and Wear Valleys NHS Trust already have a STOMP pathway and Primary care liaison nurses. The audit aims to identify a good practice with promoting STOMP in psychiatric discharge letters to primary care.

**Methods:** The audit looked at psychiatric discharges from 4 adult learning disability teams between October 2022 to October 2023 using a standard audit tool informing above standards by NICE (NG11). The data was collected by 2 authors using an Excel sheet and analysed by the lead author.

**Results:** A total of 110 of the 153 patients were prescribed psychotropics and hence included in this audit. We found 81 patients were prescribed medication for other mental health diagnoses highlighting good practice with reduced use in challenging behaviour. 102 patients (92.7%) had a documented rationale for prescribing which identifies good practice. However, only 44 (40%) and 32 (29%) patients had a strategy for review of medication and timescale for stopping medication documented. This area of concern highlighted the importance to develop recommendations to change practice.

**Conclusion:** Overall, the audit revealed poorer score in relation to documenting a strategy to review and stop psychotropic medication. The recommendations identified include increasing awareness of STOMP, promoting involvement of STOMP team in review of medications, and amending our standard psychiatric discharge templates to include prompts for timescale to stop or review medication. We also plan to review the STOMP pathway to incorporate guidance for general practitioners for when to seek specialist advice.

We hope our recommendations will improve standards regarding STOMP and patient care. We will re-audit in 6 months’ time to record the progress with above recommendations.

